# Intermetatarsal osteochondroma in a pediatric patient: a rare case of chronic foot pain and surgical resolution

**DOI:** 10.1093/jscr/rjaf636

**Published:** 2025-08-12

**Authors:** Fahad Alhuzaimi, Nouf Alabdulkarim, Mishari Alanezi, Fahad Alshayhan, Abdulrahman M Alrajhi

**Affiliations:** Department of Orthopedic Surgery, College of Medicine, King Saud University, P.O. Box 2925, Riyadh 11461, Saudi Arabia; Department of Orthopedic Surgery, College of Medicine, King Saud University, P.O. Box 2925, Riyadh 11461, Saudi Arabia; College of Medicine, King Saud University, P.O. Box 2925, Riyadh 11461, Saudi Arabia; Department of Orthopedic Surgery, College of Medicine, King Saud University, P.O. Box 2925, Riyadh 11461, Saudi Arabia; Department of Orthopedic Surgery, College of Medicine, King Saud University, P.O. Box 2925, Riyadh 11461, Saudi Arabia

**Keywords:** intermetatarsal, osteochondroma, pediatric

## Abstract

Osteochondroma involving the metatarsals is extremely rare, often presenting diagnostic and clinical challenges due to its atypical anatomical location and potential biomechanical complications. The patient, a 10-year-old boy, presented with a 2-year history of dorsal foot pain exacerbated by walking, which was unresponsive to immobilization and analgesia. Radiographic findings revealed a bony mass extending from the medial cuneiform to the first metatarsal, causing a widening between the first and second metatarsals. Given the significant effect on daily activities and persistent pain, surgical excision was performed. Intraoperatively, the lesion was consistent with osteochondroma, and complete resection was performed. His recovery was uneventful, with symptom resolution and improved functional outcomes at routine follow-up. Osteochondromas can cause pain, deformity, and restricted movement when located in weight-bearing areas. In intermetatarsal osteochondroma, surgical excision is warranted for symptomatic relief and functional improvement.

## Introduction

Osteochondroma is characterized by an abnormal bony outgrowth with a cartilaginous cap in areas of rapid bone growth, often as an incidental finding during adolescence or early adulthood [1]. Osteochondromas are most observed as solitary benign lesions; however, they may also present as multiple lesions in the context of multiple hereditary exostoses (MHE), a genetic disorder characterized by the development of numerous osteochondromas and an increased risk of malignant transformation [2]. While most osteochondromas arise from the metaphysis of long bones, foot involvement is unusual, representing unique diagnostic challenges [3]. Metatarsal involvement is rare, with such osteochondromas located mainly on a metatarsal’s plantar or dorsal surface. An intermetatarsal location is exceptionally uncommon [4].

Osteochondromas are typically asymptomatic and may go unnoticed until they cause fracture or nerve compression, at which point they can cause pain, deformity, and difficulty walking [5]. In this case report, we present an extremely rare case of osteochondroma arising between the first and second metatarsals in a 10-year-old boy, emphasizing its clinical presentation and surgical management.

## Case report

A 10-year-old boy presented with a 2-year history of right dorsal foot pain. The pain occurred when walking 50–70 meters, was not specifically associated with footwear, and occurred when walking barefoot. The patient was examined at multiple clinics and underwent trials of immobilization via cast application and analgesia. Despite conservative treatment, the patient continued to experience frequent pain and discomfort. The patient was morbidly obese and had obstructive sleep apnea. There was no family history of a genetic bone condition, and there was no evidence indicating traumatic injury or participation in high-impact sports activities.

**Figure 1:**
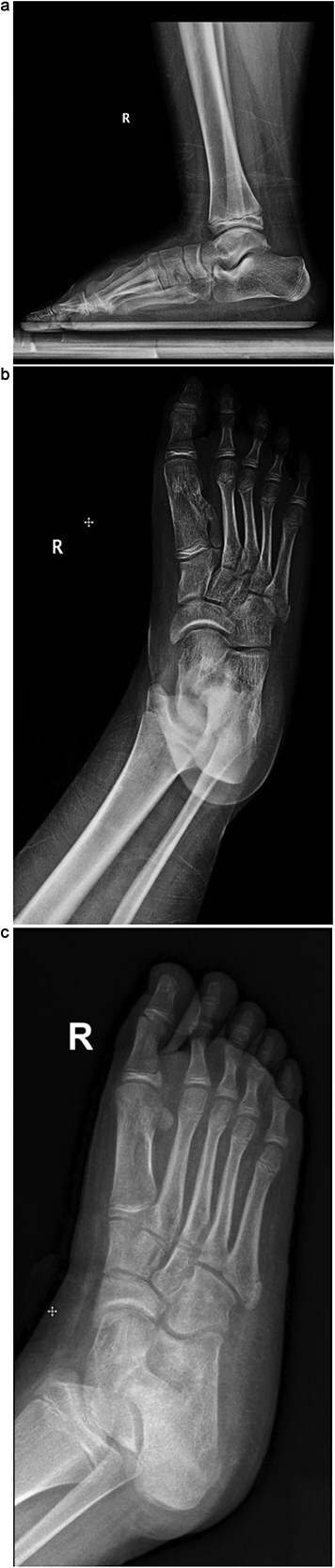
(a) Lateral radiograph of the right foot demonstrating a bony mass extending from the medial cuneiform to the first metatarsal. (b) Oblique radiograph of the right foot highlighting the abnormal bony outgrowth between the first and second metatarsals. (c) Anteroposterior radiograph showing a bony mass arising from the medial cuneiform extending toward the first metatarsal, with widening of the first intermetatarsal space.

**Figure 2:**
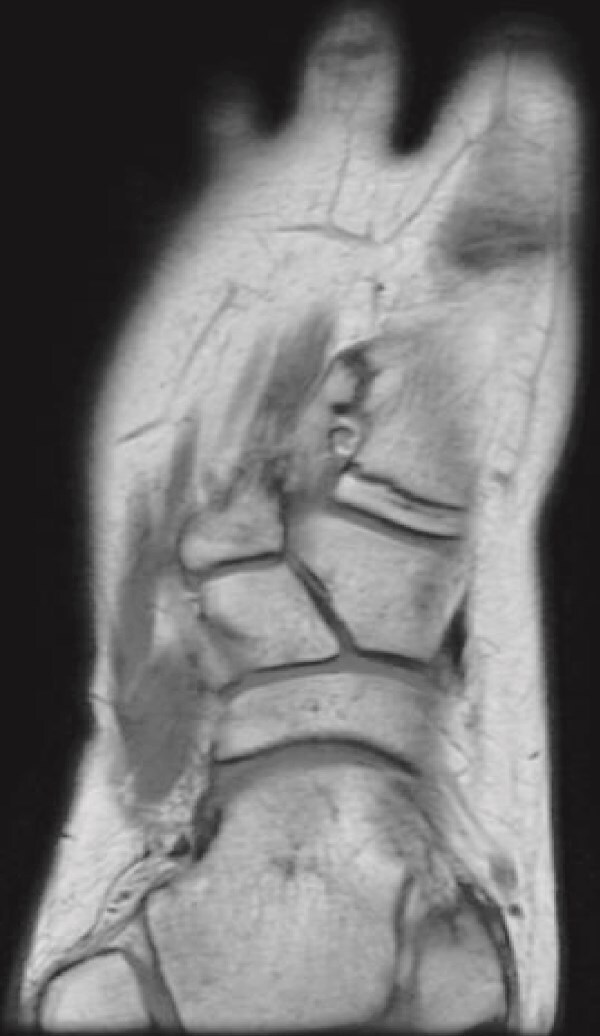
Coronal T1-weighted MRI of the right foot showing a rudimentary bone interposed between the first and second metatarsals with pseudo-articulation and ankylosis with the midshaft of the first metatarsal.

Plain radiographs revealed a bony mass extending from the medial cuneiform to the first metatarsal, with the first and second intermetatarsal space widening (Fig. 1). Magnetic resonance imaging (MRI) was performed to better characterize the mass (Fig. 2). Musculoskeletal radiologists reported the presence of an abnormal rudimentary bone that was interposed between the first and second metatarsal bones, with pseudo-articulation and ankylosis with the lateral aspect of the mid-metatarsal shaft of the hallux, causing widening and deformity, suggesting a supernumerary rudimentary metatarsal bone. An osteochondroma was also considered in the differential diagnosis, but the cartilage cap was not clearly visible. As daily activities were significantly affected, the patient’s guardian preferred surgical excision over more conservative measures, which had previously been unsuccessful.

**Figure 3:**
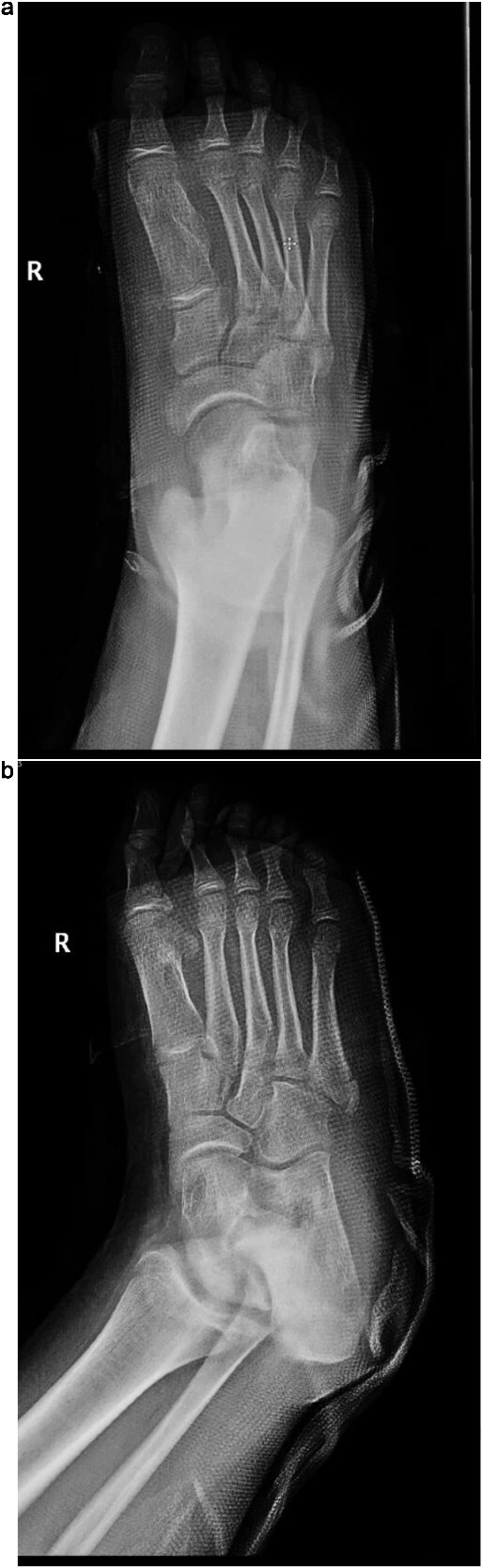
(a) Postoperative anteroposterior radiograph of the right foot demonstrating complete resection of the intermetatarsal lesion and normalization of the intermetatarsal spacing. (b) Oblique postoperative view confirming successful removal of the mass with preserved alignment of the first metatarsal and surrounding structures.

The patient was placed in the supine position for surgery. Under general anesthesia, a dorsal incision over the first metatarsal with proximal extension to the medial cuneiform was made. The subcutaneous layers were then dissected. The interval between the extensor hallucis longus and extensor hallucis brevis was identified. An exostosis extending from the medial aspect of the medial cuneiform to the medial side of the first metatarsal was identified, and its edges were defined. Resection was performed using a power saw. The Lisfranc joint was examined using dynamic testing and was deemed stable. Bone wax was applied to the resection sites, and the subcutaneous layers were approximated, followed by wound closure and dressing. Subsequently, a short back slab was applied. Postoperative radiographs are shown in Fig. 3. Postoperatively, the patient had an excellent functional outcome and was ambulating well during his routine surveillance follow-up visits at 3, 6, and 9 months.

## Discussion

Osteochondroma is a benign tumor characterized by a cartilaginous cap and a bony stalk continuous with the cortex and medulla of the underlying bone [6]. It predominantly occurs in the second and third decades, with a male predominance [7]. Osteochondromas primarily affect long bones, most commonly the femur, followed by the tibia. In contrast, involvement of the metatarsals is extremely rare, which complicates diagnosis and management [8]. When osteochondroma occurs in the foot, particularly in the metatarsals, various complications arise owing to mechanical compression on surrounding structures. These tumors can cause significant functional and cosmetic consequences. A lesion located in the intermetatarsal space may exert pressure on the first metatarsal, potentially contributing to the development of hallux abductovalgus deformity by promoting lateral deviation of the hallux [9]. Structural deformities can cause footwear discomfort, skin breakdown, and calluses. Abnormal pressure and friction lead to pain, poor shoe fit, repeated irritation, and discomfort [10, 11].

Malignant transformation of osteochondroma is a rare complication (<1% risk in solitary cases, 3% in MHEs) [12]. A sudden increase in tumor size, new onset of pain, and radiological features including irregular surface, soft tissue invasion, and >1.5 cm thickness of the cartilaginous cap are highly suggestive features for malignant transformation [13].

Diagnosing osteochondroma is mainly incidental. The pathognomonic radiological features of osteochondroma are the presence of cortical and medullary continuity from the underlying bone [14].

Management of osteochondroma involves observation of asymptomatic cases, as they are benign in nature. However, surgical excision is indicated for lesions that are causing pain or functional limitation [8].

In this case report, we describe a rare osteochondroma located between the first and second metatarsals in a 10-year-old child who underwent surgical resection. Postoperatively, the patient achieved excellent functional recovery and was ambulating well at follow-up. This case highlights the importance of considering osteochondroma in the differential diagnosis of chronic foot pain, particularly when conservative management fails.

## Data Availability

All data are available upon request to the corresponding author.
